# The effects of zinc amino acid complex supplementation on the porcine host response to *Lawsonia intracellularis* infection

**DOI:** 10.1186/s13567-018-0581-3

**Published:** 2018-09-10

**Authors:** Fernando L. Leite, Erika Vasquez, Fabio A. Vannucci, Connie J. Gebhart, Aaron Rendahl, Jerry Torrison, Adam Mueller, Nathan L. Winkelman, Zachary J. Rambo, Richard E. Isaacson

**Affiliations:** 1 Department of Veterinary and Biomedical Sciences, College of Veterinary Medicine, University of Minnesota, St. Paul, MN USA; 2 Department of Veterinary Population Medicine, College of Veterinary Medicine, University of Minnesota, St. Paul, MN USA; 3 Swine Services Unlimited Inc., Rice, MN USA; 4 Research and Nutritional Services, Zinpro Corporation, Eden Prairie, MN USA

## Abstract

*Lawsonia intracellularis* is among the most important enteric pathogens of swine and antibiotic alternatives are needed to help mitigate the negative effects of infection. Zinc is an essential trace mineral known to be crucial for maintaining intestinal barrier function and proper immune response. In this study, we investigated the porcine host response to *L. intracellularis* infection when supplemented with a zinc-amino acid complex, a form of zinc that can lead to greater bioavailability when compared to traditional inorganic forms of zinc. Our results show that a zinc-amino acid complex supplementation with a final concentration of 125 ppm of zinc in feed significantly (*p* < 0.05) decreased the number of animals with lesions and severity of lesions caused by *L. intracellularis*. Animals supplemented with the zinc-amino acid complex also exhibited a significantly (*p* < 0.05) earlier onset of seroconversion as well as an increased number of T cells in infected and non-infected intestinal tissue. This study demonstrated that this zinc-amino acid complex aids the host in responding to *L. intracellularis* infection and may be a new approach to help minimize negative effects of disease.

**Electronic supplementary material:**

The online version of this article (10.1186/s13567-018-0581-3) contains supplementary material, which is available to authorized users.

## Introduction

Zinc is an essential mineral for animals and is a critical component of many structural proteins, enzymatic processes, and transcription factors [1]. Additionally, it is known that zinc has an important role that can influence both innate and adaptive immune responses [2]. In the intestine, zinc is essential for maintaining normal barrier function and important in regeneration of injured tissue [3, 4]. Zinc can promote intestinal epithelial wound healing by enhancement of epithelial cell restitution, the initial step of epithelial wound healing [4]. In pigs, it has been demonstrated that a zinc-amino acid complex (Availa Zn) can improve trans-epithelial resistance and help maintain epithelial cell morphology in the presence of a stressor such as heat stress [5].

*Lawsonia intracellularis* is an obligate intracellular pathogen that replicates inside of intestinal, primarily ileal, enterocytes and causes the disease porcine proliferative enteropathy (PPE) [6]. This disease has two major clinical forms: porcine intestinal adenomatosis (PIA) and proliferative hemorrhagic enteropathy (PHE). PIA is the most common form of this disease and, though often characterized as mild diarrhea, is also often characterized as a subclinical manifestation leading to decreased production performance [7]. *L. intracellularis* infection leads to lesions characterized by hyperplasia of enterocytes in intestinal crypts and compromises intestinal function, leading to decreased weight gain and feed conversion efficiency [7, 8].

Considering the importance of zinc in intestinal health, it is important that it be properly supplemented. Zinc complexed with amino acids can lead to greater bioavailability as compared to inorganic forms [9, 10]. High concentrations (2000–3000 ppm) of inorganic zinc oxide have been used in swine production for treatment and prevention of diarrhea in weaning piglets but have also been associated with negative impacts such as promoting antimicrobial resistance [11, 12]. The objective of this study was to investigate the ability of zinc amino acid complex to meet the physiologic needs of the animal and its impact on the response to *L. intracellularis* infection. In this study, we compared zinc provided in an amino acid complex formulation (ZnAA) to supplementation with inorganic zinc sulfate alone. The dose used in this study was 125 ppm, which was selected because it is close to the daily requirement of swine (estimated to be around 100 ppm) [13, 14]. We additionally investigated the impact of providing ZnAA in water as compared to feed.

## Materials and methods

### Study design and animal diets

To study the impact of zinc amino acid complex provided in feed (ZnAA) (Availa Zn, Zinpro Corporation) and water (ZnAALQ) (Availa Zn LQ, Zinpro Corporation) on pigs challenged with *L. intracellularis*, a total of 4 treatment groups were used in a randomized block design. A total of 72 4-week-old pigs were randomized by weight and gender and assigned to one of four treatment groups. The pigs were of a Landrace cross to a Yorkshire female with a large white sire (Topigs-Norsvin). All pigs were negative for *L. intracellularis* infection prior to the start of the study. There were 18 pigs per treatment divided among 6 pens with three animals per pen (Table 1). The pens were equally distributed in two barns (barn A and barn B). All pigs were fed their respective diets ad libitum 21 days prior to challenge with *L. intracellularis*. Diets in all treatments were formulated to meet or exceed phase requirements [14] and were isocaloric, isonitrogenous and iso for calcium, available phosphorus levels differed only in the source of Zn and Mn. The negative and positive control groups received supplementation with 125 ppm zinc sulfate, while one of the treatment groups received the same diet but with ZnAALQ added on top of the control diet via water (diluted to provide 1.09 mg of ZnAALQ per 29.6 mL of water) while the other treatment group received ZnAA in feed at 50 ppm and another 75 ppm of zinc sulfate (a total of 125 ppm). The analyzed Zn concentration for the diet containing ZnAA was 200 mg Zn/kg and the analyzed concentration of the diet containing only ZnSO_4_ was 189.7 mg Zn/kg. All groups except the negative control group were challenged orally with a mucosal homogenate from a finishing pig with acute PHE, following a protocol previously described [15]. Briefly, to obtain the homogenate, mucosa was scraped from the small intestine of a pig affected with PPE lesions, and suspended at a 1:1 ratio in sucrose-phosphate-glutamate (SPG) buffer, pH 7.0, which contained sucrose (0.218 M), monobasic potassium phosphate (0.0038 M), dipotassium phosphate (0.0072 M), and l-glutamic acid (0.0047 M) [15]. The pig that was used for the challenge homogenate was screened for other known swine pathogens and none were found. Animals were challenged with a dose of about 8.0 × 10^7^ *L. intracellularis* organisms by oral gavage at 7 weeks of age. Challenge dose was estimated by real time PCR. Groups of 6 pigs were randomly selected from each group of pigs per treatment and were euthanized at 14, 21 and 28 days post-challenge by electrocution.

**Table 1:** **Animal treatments**

Treatment	Pens/treatment	Pigs/pen	Pigs/treatment	Zn level (ppm) and source	Challenge
Positive control	6	3	18	125, SO_4_	Yes
ZnAA in feed	6	3	18	75 SO_4_ + 50 Availa ZN	Yes
ZnAALQ in water	6	3	18	125 SO_4_ + Availa ZN LQ	Yes
Negative control	6	3	18	125, SO_4_	No

### Animal production measurements

To investigate the impact of zinc supplementation with ZnAA and ZnAALQ on the production performance of animals when infected with *L. intracellularis*, the average daily weight gain (ADG), average daily feed intake (ADFI), gain to feed ratio (G:F), and water disappearance were calculated. Animals were weighed weekly and recording of feed and water provided was recorded daily. Water disappearance was calculated by weighing water volumes every time water was provided to estimate the amount of water that had disappeared within a timeframe. ADG, ADFI, and water disappearance were calculated by measuring the difference of animal weight and feed and water consumed between each week dividing the difference by 7 to obtain daily averages for each week of the trial. For ADFI and water disappearance, the value was then divided by the number of animals per pen to obtain a per animal average. This calculation still considers the pen as the experimental unit. Gain to feed ratio was obtained by dividing ADG by ADFI values.

### Clinical observations

Clinical signs were recorded throughout the experimental period and animals were evaluated for demeanor score, abdominal score and fecal score. All scores used for evaluation varied from zero to three with increasing severity of clinical signs. Demeanor scores ranged from zero to three with 0 representing normal animal behavior and 3 being severely depressed/partially recumbent. Abdominal scores of zero represented normal and scores of three represented severely gaunt animals. A fecal score of zero represented no diarrhea, a score of one represented semi-solid/not formed feces, a score of two represented watery stool with less than 50% of water and a score of three represented profuse projectile diarrhea.

### Gross and microscopic pathology evaluation

At 14, 21 and 28 days post-infection (dpi) groups of animals (*n* = 6) were randomly selected from each treatment group and were euthanized. Post-mortem examination of the ileum, jejunum, cecum and colon of all animals was carried out to identify the presence of gross pathologic change including those characteristic of PPE. Each lesion was scored as either mild (score of 1), moderate (score of 2) or severe (score of 3). For histopathology and evaluation of microscopic lesions characteristic of PPE, a section of the ileum proximal to the ileocecal valve was collected from all animals. Tissues were immediately fixed in formalin and then embedded in paraffin, and cut. The terminal ileum was selected for sampling since it is the most consistent site of *L. intracellularis* infection and lesions [16]. Histological evaluation was performed after staining with hematoxylin and eosin (HE) and immunohistochemistry (IHC) was performed using rabbit anti-*L. intracellularis* specific polyclonal antibody to measure the extent of proliferative lesions and to quantify the amount of *L. intracellularis* present in the tissues, respectively. The presence of *L. intracellularis*-specific antigen was measured blindly with a five-grade IHC scoring scale (grade 0 equal to absence of antigen in tissue; and grade 4 equal to all enteric crypts containing antigen) as previously described [17]. The extent of lesions was measured on a 3 point grading scale representing the distribution of crypt dysplasia with 1 assigned for focal lesions, 2 for multifocal lesions, and 3 for diffuse lesion distribution.

### Evaluation of B cell distribution and T cell quantification

Since zinc is known to affect the immune system, we quantified T cell and B cell populations to determine if there were potential changes in immune responses at the site of infection based on cell population changes. For this, antibodies against CD3 (T cell) and CD79a (B cell) were used. After blocking nonspecific binding sites with 10% normal goat serum for 15 min, slides were incubated with primary antibodies for 30–45 min at room temperature. Slides were then rinsed with tris-buffered saline with 0.05% Tween 20 and incubated with horseradish peroxidase conjugated secondary antibodies to allow visualization of cells in tissue. The slides were then observed at 100× and 400× magnification using a light microscope. The distributions of B cells were scored on a 1–4 scale as follows: a focal distribution was given a score of 1, a multifocal distribution given a score of 2, a multifocal-to-diffuse distribution given a score of 3 and a diffuse distribution given a score of 4. To quantify T cells, 60 crypts per animal were selected, 30 infected with *L. intracellularis* and 30 non-infected. The total number of T cells present in each crypt was measured and averaged per animal.

### Antibody response and fecal shedding

To investigate the antibody response of the pigs to *L. intracellularis*, serum samples were collected by venipuncture of the cranial vena cava at 14, 21 and 28 dpi. Serum samples were serially diluted from 1:30 to 1:1920 in phosphate-buffered saline containing 2.5% fetal bovine serum, 1.0% rabbit serum and 0.08% tween 80 and screened for antibodies against *L. intracellularis* using the immunoperoxidase monolayer assay (IPMA) [18]. To measure shedding of *L. intracellularis*, fecal samples were collected at the same time points and DNA was extracted. Real time PCR was then used to estimate the quantity of *L. intracellularis* using standardized methods at the University of Minnesota Veterinary Diagnostic Laboratory.

### Statistical testing

Statistical tests were performed using R [version 3.3.3 (2017-03-06)] using a mixed model and differences in lsmeans evaluated for statistical significance. The pen was considered the experimental unit for all assessments. *p* values below 0.05 were considered significant. Trends were described as statistical results with *p* values below 0.1. To compare samples from positive and negative animals for seroconversion, gross lesions, IHC and HE lesions, the N − 1 Chi squared test was used.

## Results

### Clinical measurements of disease

There was only one animal with a fecal score above zero following challenge with *L. intracellularis* and that animal had a score of one indicating that it had a loose stools. This occurred at 19 dpi and the animal was in the positive control group. All animals in the negative control group remained negative for *L. intracellularis* infection. There were no cases of diarrhea or clinical signs characteristic of PPE throughout the experimental period, thus any disease induced was subclinical.

### Production measurements

Average daily gain varied from an overall average of 0.56 kg per day at day 14 of the trial to 0.92 kg per day at the last time point (28 dpi) regardless of treatment (Table 2). There were no statistical differences between treatments when comparing at a single time point.

**Table 2:** **Average daily gain**

Treatment	−7 dpi	0 dpi	7 dpi	14 dpi	21 dpi	28 dpi
Positive control	0.58	0.69	0.83	0.74	0.87	0.91
Negative control	0.54	0.66	0.80	0.84	0.92	1.03
ZnAA LQ	0.58	0.77	0.86	0.78	0.90	0.94
ZnAA	0.55	0.73	0.84	0.75	0.88	0.80
Standard error	0.04	0.04	0.04	0.04	0.005	0.06

Average daily gain was calculated in kg by measuring the difference of animal weight between each week of the experiment dividing the difference by 7 to obtain daily averages for each week of the experiment. Reported are the lsmeans, and the standard error of each time point. No significant differences were found between treatments but only between time points (*p* < 0.05).

For average daily feed intake, there was a barn effect as barn B had a higher overall feed intake average of 1.35 kg compared to 1.23 kg in barn A (*p* < 0.05). There were no trends nor statistical differences observed in ADFI when comparing treatments (Additional file 1). Based on average daily feed intake and analyzed Zn concentration, on average animals consumed 252.85 mg of total zinc daily.

There was a trend for the group treated with ZnAA in feed to have a lower gain to feed ratio compared to the negative control group at 28 dpi (Additional file 2). These ratios were of 0.438 and 0.502, respectively (*p* < 0.1). Similar to ADG and ADFI, there were no statistical differences between treatments. Again, there was a difference between both barns, which was of 0.051 averaged over all time points (*p* < 0.05).

Water disappearance ranged from 1.46 L on day 7 of the study (−14 dpi) to 3.81 L on average per animal at the last time point of the study (28 dpi) (Additional file 3). There were no barn effects or statistical differences between treatments. Statistical significance was only found comparing different time points overtime. Based on water disappearance, animals provided zinc in water consumed an additional 56.14 mg of ZnAALQ on day 7 and 139.53 mg on day 28 dpi via water disappearance.

### Gross lesions

**Figure 1:**
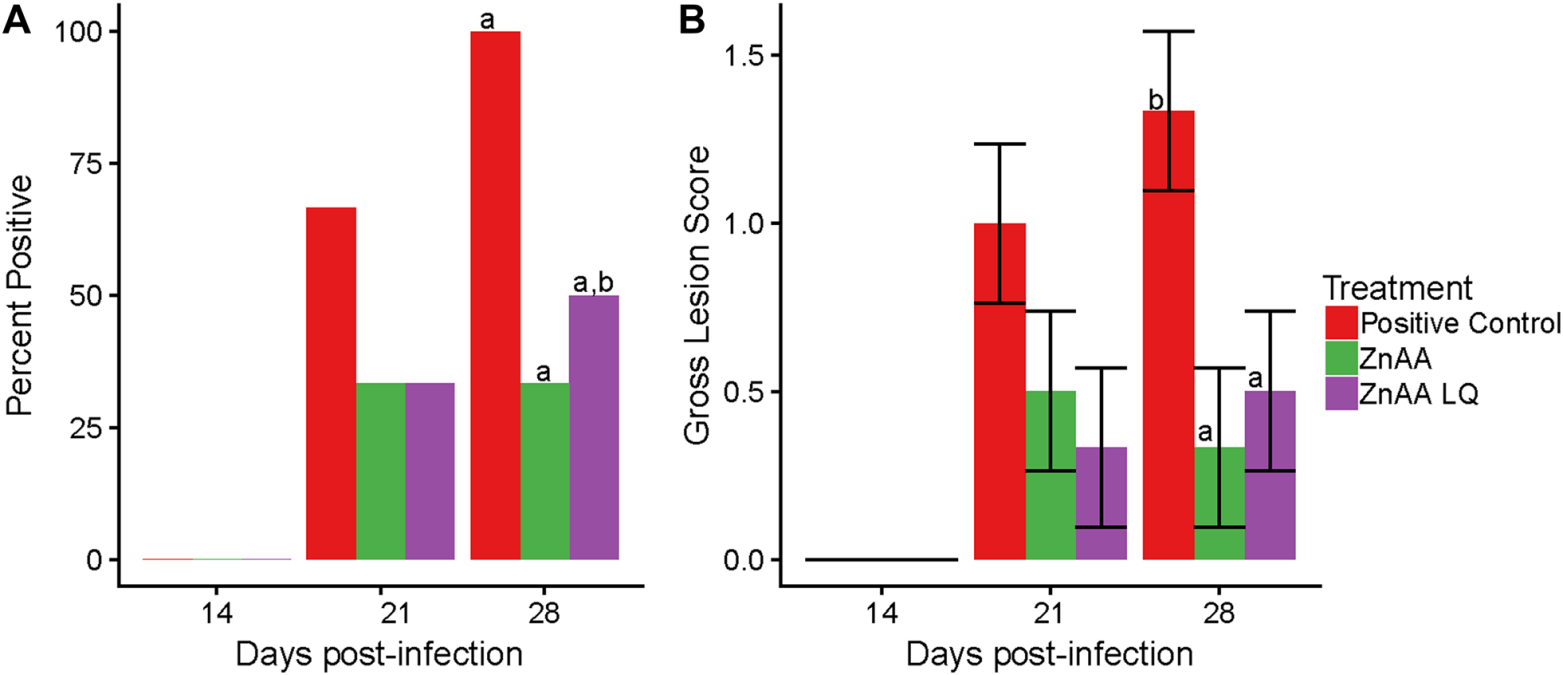
**Evaluation of gross lesions.** At 14, 21 and 28 dpi gross porcine proliferative enteropathy (PPE) lesions were evaluated and assigned the scores of either mild (score of 1), moderate (score of 2) or severe (score of 3). **A** Percent of animals with gross PPE lesions evaluated at necropsy. **B** Lesion score evaluated at necropsy. Different letters indicate statistical significance (*p* < 0.05), error bars represent the standard error.

No pigs had gross lesion scores consistent with PPE at 14 dpi. On both 21 and 28 dpi, pigs in both the ZnAA and ZnAALQ treatment groups had fewer animals with gross lesions compared to pigs in the positive control group (Figure 1A). At 21 dpi, 33% of animals that received either ZnAA treatment had gross lesions (2 of 6 animals) while 66% of animals in the positive control group had gross lesions (4 of 6 animals). At 28 dpi, the ZnAA group had significantly less animals with lesions (*p *< 0.05) compared to the positive control group with 33% (2 of 6) and 100% (6 of 6) of animals with lesions, respectively. At this time point, the ZnAALQ treatment led to a trend (*p* < 0.1) in reduction in the number of animals with gross lesions compared to the positive control group having 50% of animals with lesions (3 of 6). Both ZnAA and ZnAALQ treatments led to a significant (*p* < 0.05) reduction in lesion severity at 28 dpi with the average lesion score being 1.33 in the positive control group compared to 0.33 in the ZnAA and 0.5 in the ZnAALQ group (Figure 1B).

### Immunohistochemistry and microscopic lesions

**Figure 2:**
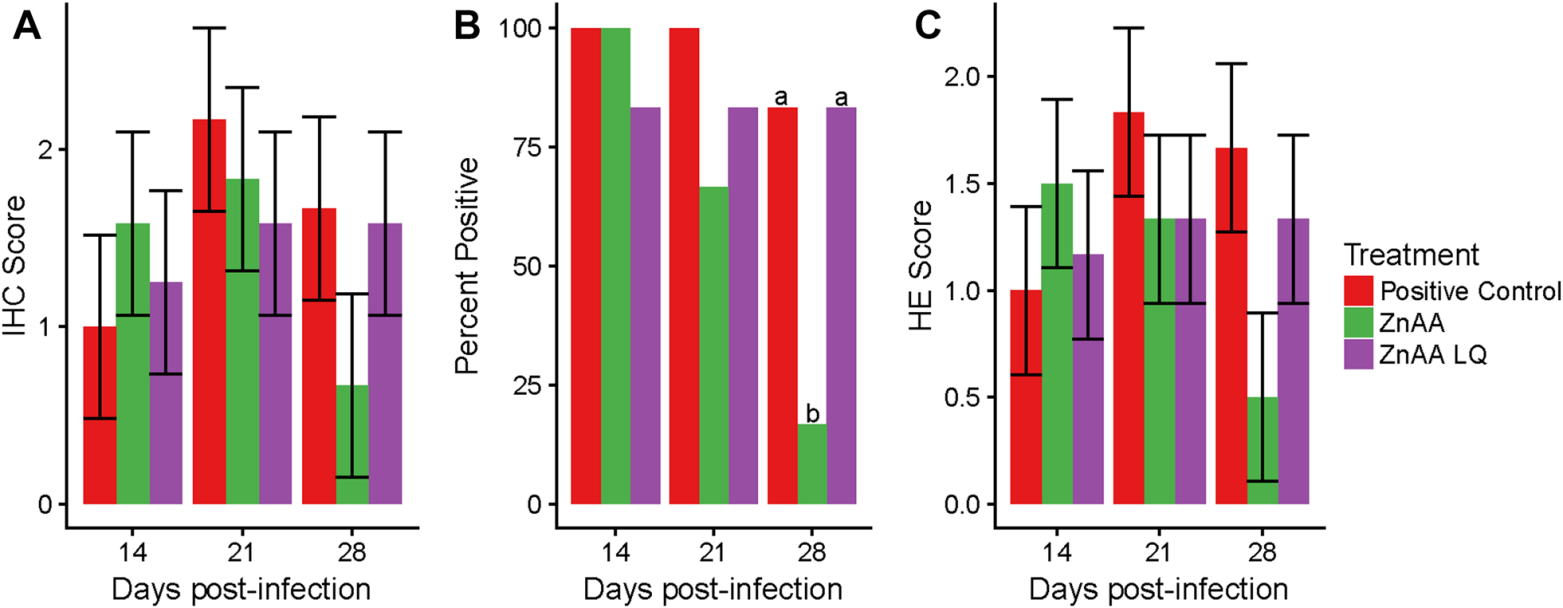
**Evaluation of immunohistochemistry and microscopic lesions.** At 14, 21 and 28 dpi immunohistochemistry (IHC) was performed to semi-quantify *L. intracellularis* in tissue and hematoxylin and eosin (HE) stain was performed to evaluate microscopic lesions. IHC scores used were of 0: absence of antigen in tissue; 1: 0–25%, 2: 25–50%; 3: 50–75%; 4: 75–100% of enteric crypts containing *L. intracellularis* antigen. HE scores used were of 1: focal lesions; 2: multifocal lesions; 3: diffuse lesions. **A** IHC score per time point after infection. **B** Percent of animals with microscopic lesions evaluated by HE. **C** HE score per time point after infection. Different letters indicate statistical significance (*p* < 0.05), error bars represent the standard error.

To estimate the amount of *L. intracellularis* present in the intestinal tissue, IHC was performed. All treatment groups except the negative control group had *L. intracellularis* antigen present in tissue at 14, 21 and 28 dpi. The IHC score for both the ZnAA and the positive control groups peaked at 21 dpi while the ZnAALQ group had similar IHC scores at 21 and 28 dpi. At 28 dpi, the group treated with ZnAA had the lowest IHC score with an average score of 0.67 compared to the positive control and ZnAALQ groups which had scores of 1.6 and 1.58, respectively (Figure 2A).

In addition to determining the extent of infection by measuring bacterial presence, the extent of microscopic lesions was determined using paraffin embedded samples that were stained with HE. Trends observed in microscopic lesion severity were similar to those for IHC score (Figure 2C). The number of animals with microscopic lesions after receiving ZnAA in feed was significantly reduced (*p* < 0.05) compared to the number of animals in the positive control and ZnAALQ treatment groups with lesions. At 28 dpi, only 17% (1 of 6) of animals in the ZnAA group had lesions compared to 83% (5 of 6) of pigs in the positive control and ZnAALQ groups, respectively (Figure 2B).

### Quantification of *L. intracellularis* shedding and serum antibody response

Since *L. intracellularis* antigen in tissues was reduced in pigs given with ZnAA, we next investigated if ZnAA supplementation reduced shedding of *L. intracellularis* in feces. Therefore, real time PCR to detect *L. intracellularis* in feces was performed on samples collected at 14, 21 and 28 dpi. No differences in fecal shedding were observed between treatment groups (Additional file 4). A Ct value below 35 was considered a positive result. There was a barn effect as barn B had an average Ct value for all treatments and time points of 27 while barn A had an average Ct of 30.2 (*p* < 0.05). These values correspond to approximately 3 × 10^4^ and 5 × 10^5^ organisms per gram, respectively. There were no statistical differences between treatments but only between sampling days.

**Figure 3:**
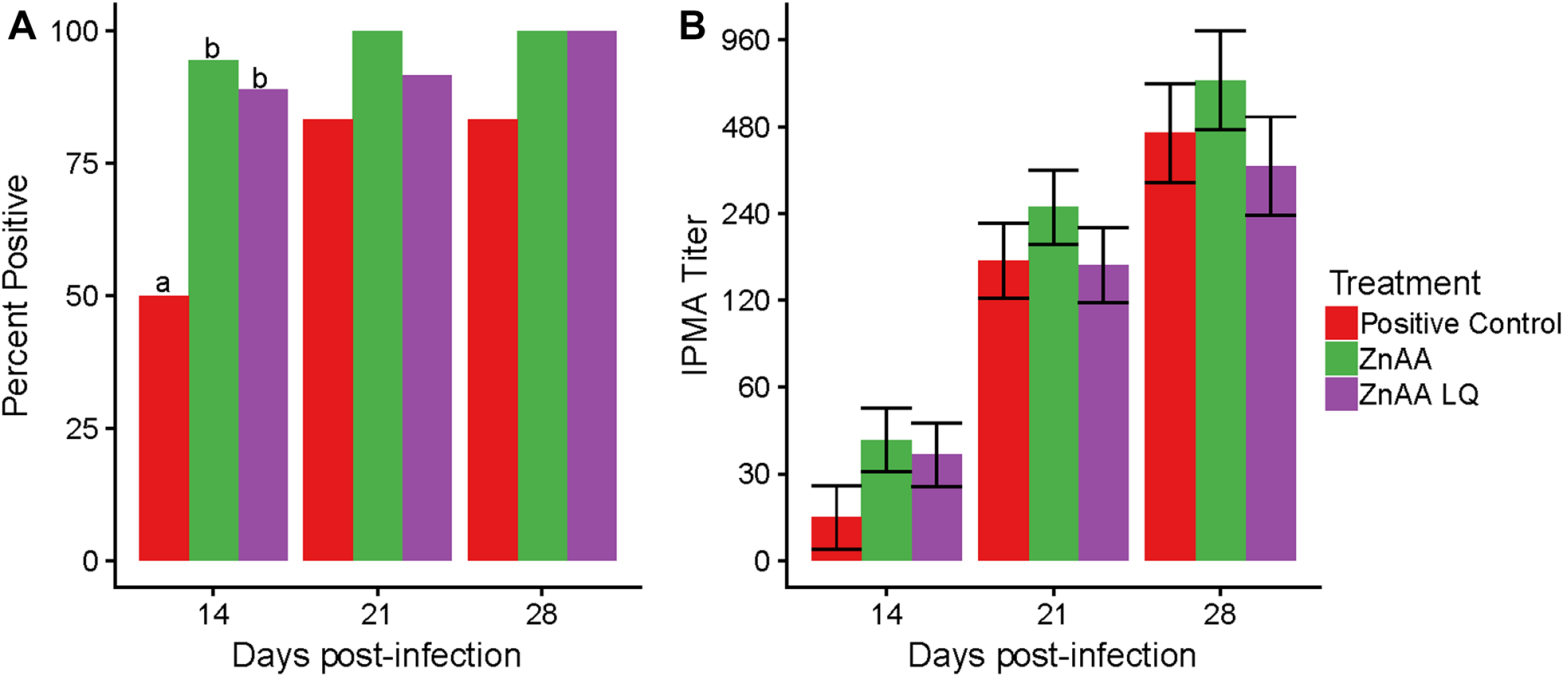
**Evaluation of serum antibody responses in animals.** At 14, 21 and 28 dpi, the IPMA assay was used to quantify *L. intracellularis* specific antibodies in serum of animals. **A** Percent of animals with antibodies in serum against *L. intracellularis*. **B** IPMA titer per treatment at different timepoints post-infection. Different letters indicate statistical significance (*p* < 0.05), error bars represent the standard error. No significant differences were found in IPMA titers between treatments but only between time points (*p* < 0.05).

Since ZnAA reduced mucosal lesions, we wondered if ZnAA had an impact on the immune response to infection. We evaluated humoral immune response by testing antibodies against *L. intracellularis* in serum at 14, 21 and 28 dpi. At 14 dpi, the ZnAA and ZnAALQ treatments led to a significant increase in the number of animals with antibodies against *L. intracellularis* demonstrating that they seroconverted at an earlier time point than the positive control group (*p* < 0.05) (Figure 3A). This difference was of 94% seroconversion in the ZnAA group (17 of 18 animals) and 89% seroconversion (16 of 18 animals) in the ZnAALQ group compared to 50% seroconversion in the positive control group (9 of 18 animals). Statistically significant differences in antibody titers were not observed between the treatment groups when comparing different days to each other (Figure 3B), thus demonstrating that treatment with ZnAA only reduced the time to seroconversion but did not affect the magnitude of the response. No animals seroconverted in the negative control group.

### Quantification of B Cells and T cells

**Figure 4:**
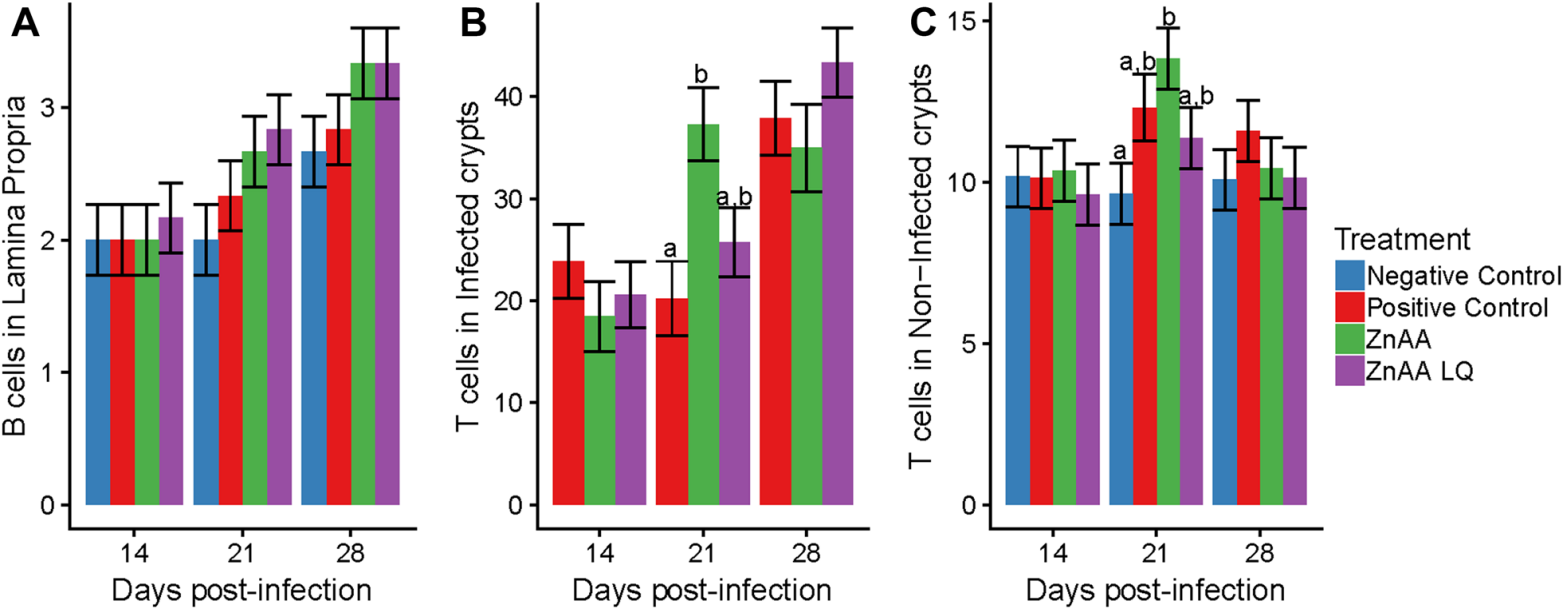
**Evaluation of B and T cells in ileum tissue.** At 14, 21 and 28 dpi, both B cells and T cells were evaluated in the terminal ileum of all animals. The distribution of B cells was evaluated and given the score of either 1: focal distribution, 2: multifocal distribution, 3: multifocal-to-diffuse distribution, 4: diffuse distribution. T cells were counted in 30 infected and 30 non-infected crypts per animal. **A** Distribution of B cells in lamina propria. **B** Number of T cells per infected crypt. **C** Number of T cells per non-infected crypt. Error bars represent standard error. Different letters indicate statistical significance (*p* < 0.05).

Since ZnAA had an impact on humoral immunity, next we wanted to evaluate changes to the population of B cells and T cells at the site of infection. Treatment with either ZnAA or ZnAALQ led to a significant increase in the distribution of B cells in the lamina propria of the ileum (*p* < 0.05) over time, while pigs in the positive control group showed a trend toward B cell increases in the ileal lamina propria (*p* < 0.1) (Figure 4A).

To quantify T cells, both crypts infected with *L. intracellularis* and non-infected crypts were evaluated. Among infected crypts, the ZnAA group had significantly more T cells than the positive control group (*p* < 0.05) at 21 dpi and a trend of higher numbers of T cells compared to the ZnAALQ group (*p* < 0.1) (Figure 4B). The ZnAA group was the only one that had a significant increase in T cells of non-infected crypts compared to the negative group at 21 dpi (*p* < 0.05) (Figure 4C). No other significant differences were found when comparing T cells of non-infected crypts.

## Discussion

This study was performed to investigate the effect of zinc amino acid complex supplementation on the host immune response to *L. intracellularis* infection and investigate its potential effects in animal production performance during infection. Significant differences were observed using traditional methods of disease measurement including gross and microscopic PPE lesions. ZnAA given in feed or ZnAALQ given in water both led to an overall reduction in the number of animals with gross lesions and a significant decrease in the severity of lesions at 28 dpi (*p* < 0.05) (Figure 1). In terms of microscopic lesions, ZnAA given in feed led to a large and significant (*p* < 0.05) decrease (from 83 to 17%) in the number of animals with lesions at 28 dpi (Figure 2B). This was also the treatment with the least amount of *L. intracellularis* present in ileum tissue at 28 dpi, suggesting earlier resolution of lesions (Figure 2A).

In the normal course of experimental infection, the initial detection of *L. intracellularis* inside of enterocytes occurs at approximately 5 dpi, and microscopic lesions are detected around 10 dpi [7, 19]. Disease generally peaks around 21 dpi and at approximately 28 dpi the resolution of lesions generally occurs [7, 19]. The finding that zinc amino acid complex supplementation leads to decreased lesion severity at 28 dpi compared to positive control may indicate that it leads to a shorter disease course and/or a more efficient host response leading to enhanced clearance of infection. This is especially true since animals in the positive control group still had severe lesions at 28 dpi. The observed earlier seroconversion post-challenge is consistent with this conclusion. An earlier onset of disease (up to a week) leading to earlier resolution of lesions cannot be ruled out; however.

There were no statistically significant differences between treatments in ADG, ADFI, G:F ratio or water disappearance. Two factors that likely contributed to this were the lack of a clinical impact on overt disease since the challenge dose of *L. intracellularis* only induced mild disease and the low number of animals used in this trial. The dose given to animals was chosen to induce subclinical disease to better mimic field conditions. Subclinical *L. intracellularis* infection does reduce production performance but may require a larger number of animals to reach statistical significance in a study [8, 20]. Although significant differences in performance were not observed with challenge, *L. intracellularis* infection was successfully reproduced as confirmed by macroscopic and microscopic lesions, *L. intracellularis* specific IHC in ileum tissue and antigen specific serum antibody response. Two trends were identified indicating that ZnAA in feed may lead to decreased weight gain and gain to feed ratio. These differences were observed at the time point with the least number of animals per group and should be confirmed with a greater number of animals.

The World Health Organization recommends zinc supplementation for the treatment of diarrhea in children in developing countries. Zinc has been shown to reduce both the duration and severity of diarrhea [21]. This effect includes diarrhea caused by several infectious agents including rotavirus, *Vibrio cholerae*, *Shigella*, *Salmonella* and protozoa such as *Cryptosporidium parvum* and *Giardia lamblia*. *L. intracellularis* infection has not been described in humans. While diets in this study were not deficient in zinc according to the National Research Council (NRC) [14], and contained similar zinc levels in feed, we hypothesized that supplementation with a more bioavailable form of zinc would have a greater effect on the host immune response as compared to zinc sulfate alone. Although clinical disease (i.e. diarrhea) was not observed in this study, ZnAA supplementation still had a positive role in the host response to infection.

The mechanism(s) responsible for zinc attenuation of enteric disease is not completely understood although zinc is known to have an impact on the immune system [2, 21]. Similar to the results seen in this study, Rahman et al. found that children infected with *Shigella flexneri* also had significant increase in seroconversion to *S. flexneri* when given a zinc supplement (20 mg zinc acetate daily) [22]. A reduction of pathogen load and earlier onset of humoral response was also observed when comparing zinc supplemented to zinc deficient mice infected with *Trypanosoma musculi* [23]. This may have been one of the factors that led to the observation of beneficial effects of zinc amino acid complex in decreasing lesion severity and decreasing the amount of *L. intracellularis* present in ileum tissue. Both ZnAA and ZnAALQ led to a significant increase in the number of animals that seroconverted at 14 dpi (Figure 3).

The effects that zinc can have on antibody- and cell-mediated immune responses in both humans and animals have been recognized for a long time [24]. Dietary zinc level has been shown to be associated with a host’s capacity to produce antigen-specific antibody [25]. Thus it is not surprising that the ZnAA treatments led to a faster humoral immune response. One of the effects of inadequate zinc levels in the body is lymphopenia [24, 25]. Potentially, this mechanism of increased lymphocytes with zinc supplementation could be related to the observation that both ZnAA treatments led to a significant rise in the distribution of B cells in ileum tissue from 14 to 28 dpi. This increase was higher than the one observed in animals that did not receive ZnAA (Figure 4B). Adequate zinc levels have also been shown to have a positive effect on T cell function and proliferation [26]. ZnAA led to a significant increase in the number of T cells at 21 dpi in both infected and non-infected tissue (*p *< 0.05) (Figures 4B and C). This increase likely also contributed to the superior ability of the animals in this treatment group to resolve lesions and remove *L. intracellularis* from infected tissue.

Both the positive control group and the ZnAA in feed group received the same calculated concentration of zinc in their diet (125 ppm, Table 1) and assayed zinc values were similar. Yet these treatments led to very different responses in pigs when challenged with *L. intracellularis*. Zinc is a trace mineral that is not efficiently stored in the body, thus daily intake is required to achieve adequate steady-state concentrations to support all its functions [2]. It is likely that the zinc amino acid complex led to superior supplementation than zinc alone in the sulfate form, as has been suggested in previous studies [9, 10].

Overall, this study demonstrated the impact of zinc amino acid complex supplementation on the host response to *L. intracellularis* infection. Decreases in gross and microscopic lesions were observed at 28 dpi, along with increased seroconversion at 14 dpi compared to the group that was challenged and supplemented with inorganic zinc alone. Although benefits were observed only at specific timepoints, these results indicate that zinc supplementation in the form of amino acid complex provided in water or in feed aid the pig immune response to infection and lead to decreased number and severity of PPE lesions. Given the ban of antibiotics as growth promoters in the US and Europe, alternatives to improve animal growth including effective ways to prevent or treat certain infections are needed. The results presented here demonstrate that the possible usage of zinc amino acid complexes may be one alternative.

## Additional files

**Additional file 1.** **Average daily feed intake measured in different treatment groups throughout experiment.** The amount of feed (in Kg) consumed per animal was calculated by measuring the difference of feed consumed between each week dividing the difference by 7 to obtain daily averages for each week of the trial. Numbers were divided by the number of animals per pen to obtain an average per animal. Error bars represent standard error. No significant differences were found between treatments but only between time points (*p* < 0.05).

**Additional file 2.** **Gain to feed ratio measured in different treatment groups throughout experiment.** Gain to feed ratio was obtained by dividing average daily gain by average daily feed intake values. Error bars represent standard error. No significant differences were found between treatments but only between time points (*p* < 0.05).

**Additional file 3.** **Water disappearance measured in different treatment groups throughout experiment.** Recording of water provided was performed daily. Water disappearance was calculated by measuring the difference of water consumed between each week dividing the difference by 7 to obtain daily averages for each week of the trial. This value was then divided by the number of animals per pen to obtain a per animal average. Error bars represent standard error. No significant differences were found between treatments but only between time points (*p* < 0.05).

**Additional file 4.** **PCR CT values measured in different treatment groups throughout experiment.** Real time PCR was used to estimate the quantity of *L. intracellularis* shed in feces at different time points post-infection. Error bars represent standard error. No significant differences were found between treatments but only between time points (*p* < 0.05).

## Abbreviations

PPE: porcine proliferative enteropathy
PIA: porcine intestinal adenomatosis
PHE: proliferative hemorrhagic enteropathy
ZnAA: zinc amino acid complex formulation provided in feed
ZnAALQ: zinc amino acid complex formulation provided in water
ADG: average daily weight gain
ADFI: average daily feed intake
F:G: feed to gain ratio
HE: hematoxylin and eosin
IHC: immunohistochemistry
IPMA: immunoperoxidase monolayer assay
dpi: days post-infection
NRC: National Research Council
