# Supplementary figures and images for: The effects of zinc amino acid complex supplementation on the porcine host response to Lawsonia intracellularis infection
Source: Vet Res. 2018 Sep 10;49:88. doi: 10.1186/s13567-018-0581-3 (PMC6131730; doi:10.1186/s13567-018-0581-3)

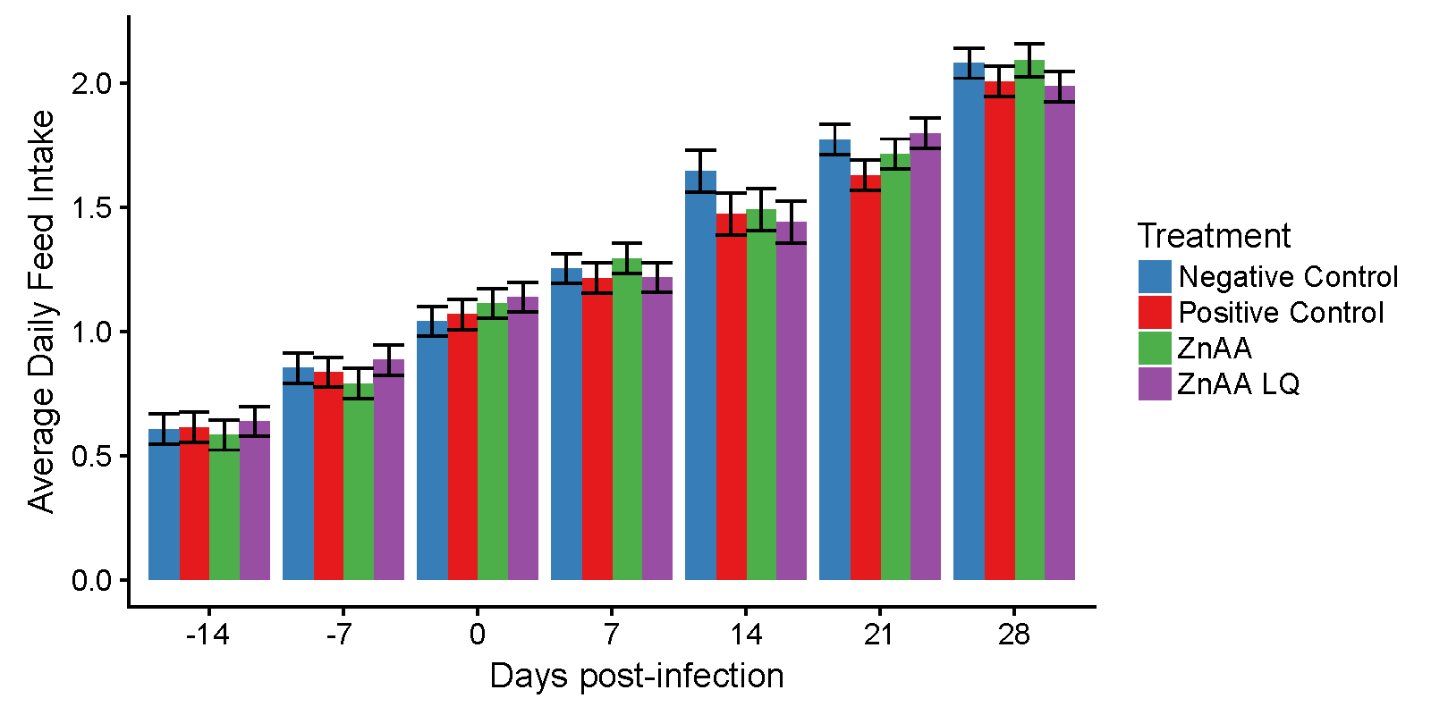

Supplement: Supplementary file 1 — Additional file 1. Average daily feed intake measured in different treatment groups throughout experiment. The amount of feed (in Kg) consumed per animal was calculated by measuring the difference of feed consumed between each week dividing the difference by 7 to obtain daily averages for each week of the trial. Numbers were divided by the number of animals per pen to obtain an average per animal. Error bars represent standard error. No significant differences were found between treatments but only between time points (p < 0.05). [file 13567_2018_581_MOESM1_ESM.docx]

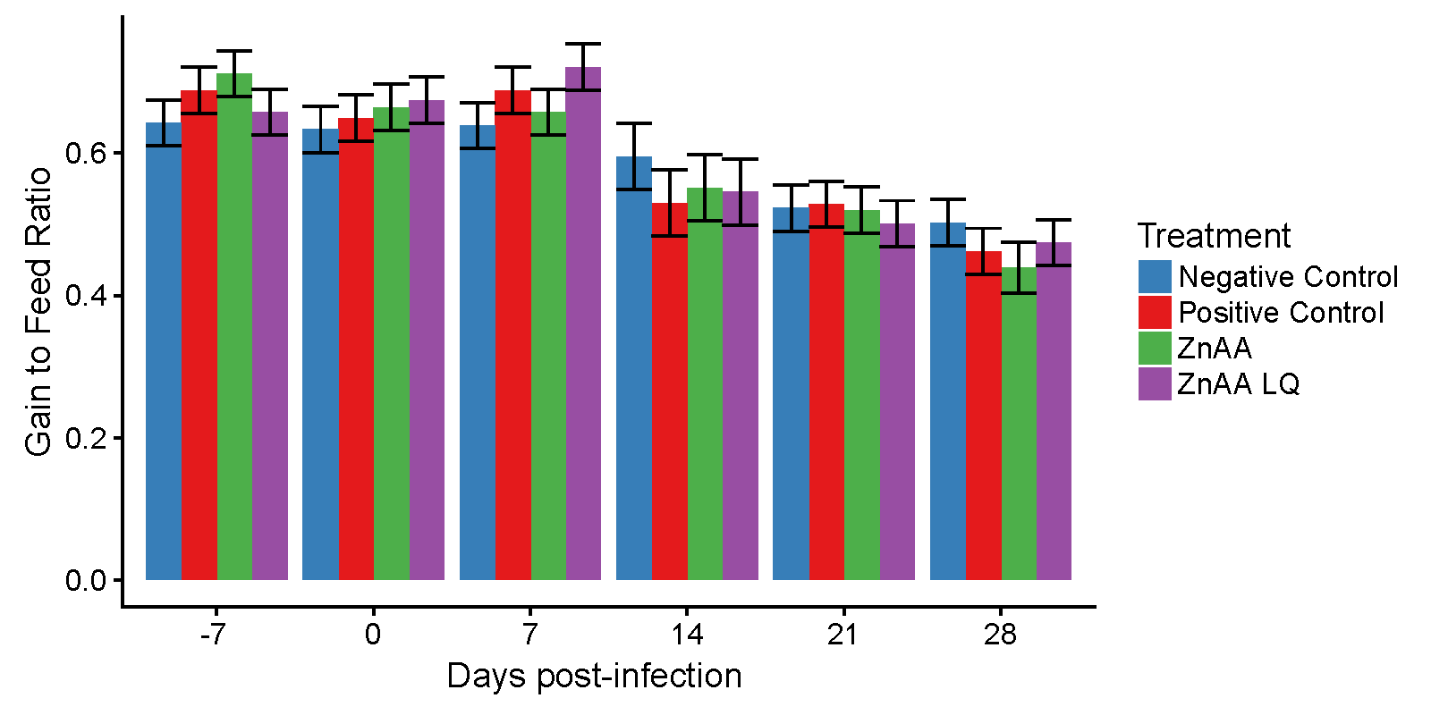

Supplement: Supplementary file 2 — Additional file 2. Gain to feed ratio measured in different treatment groups throughout experiment. Gain to feed ratio was obtained by dividing average daily gain by average daily feed intake values. Error bars represent standard error. No significant differences were found between treatments but only between time points (p < 0.05). [file 13567_2018_581_MOESM2_ESM.docx]

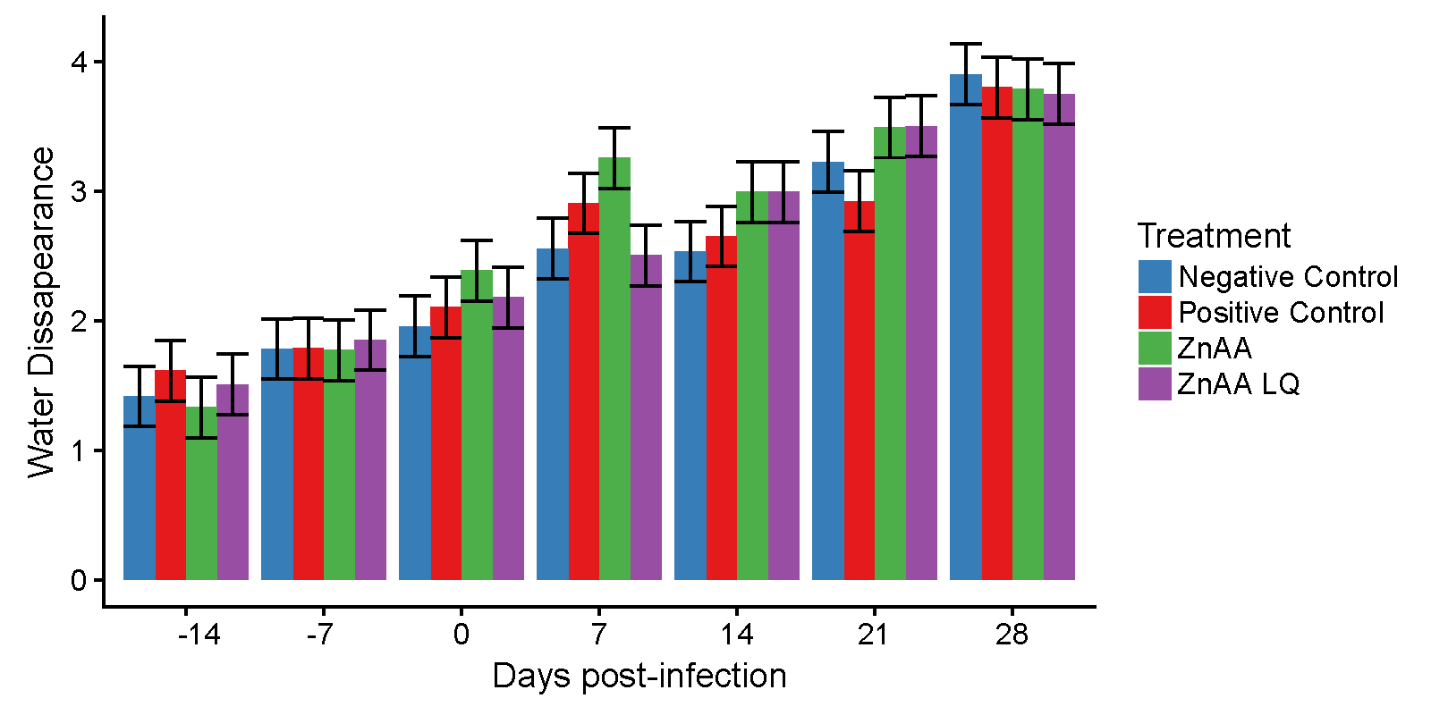

Supplement: Supplementary file 3 — Additional file 3. Water disappearance measured in different treatment groups throughout experiment. Recording of water provided was performed daily. Water disappearance was calculated by measuring the difference of water consumed between each week dividing the difference by 7 to obtain daily averages for each week of the trial. This value was then divided by the number of animals per pen to obtain a per animal average. Error bars represent standard error. No significant differences were found between treatments but only between time points (p < 0.05). [file 13567_2018_581_MOESM3_ESM.docx]

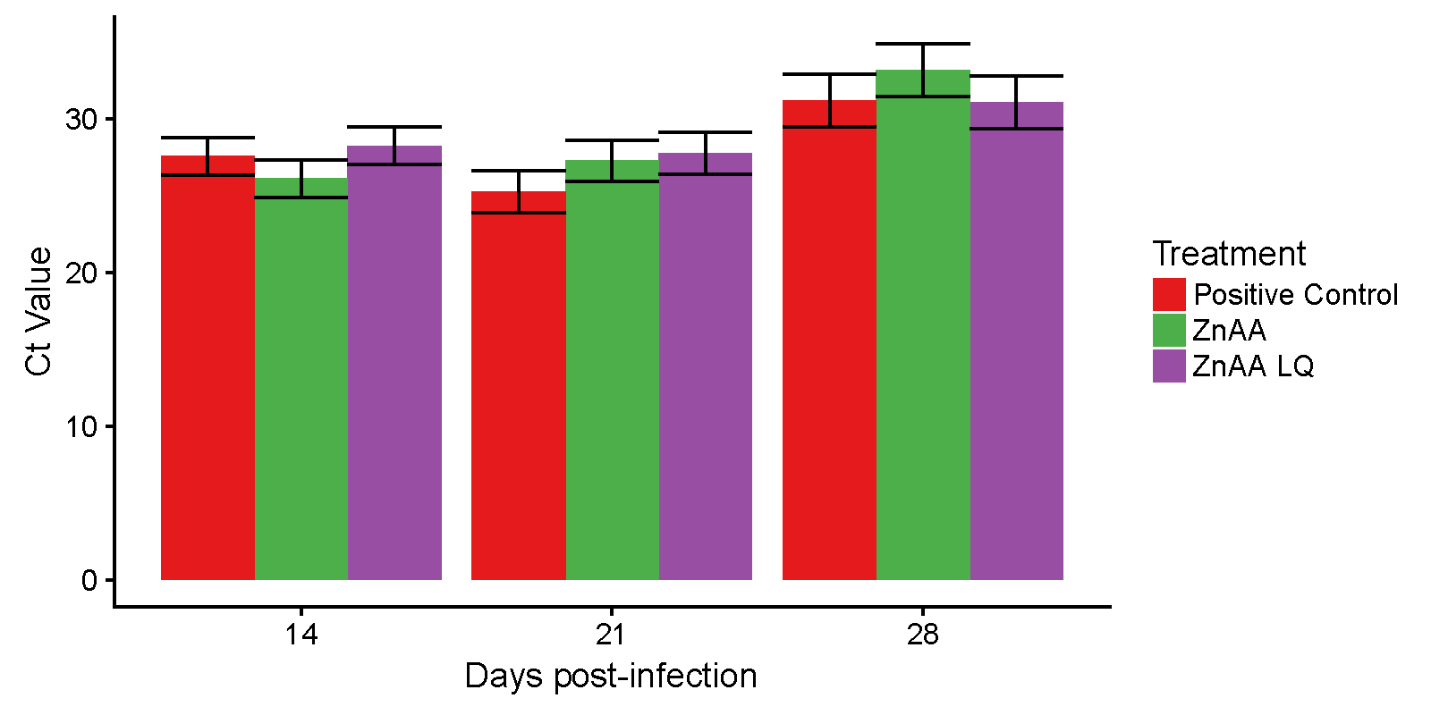

Supplement: Supplementary file 4 — Additional file 4. PCR CT values measured in different treatment groups throughout experiment. Real time PCR was used to estimate the quantity of L. intracellularis shed in feces at different time points post-infection. Error bars represent standard error. No significant differences were found between treatments but only between time points (p < 0.05). [file 13567_2018_581_MOESM4_ESM.docx]
